# The Sorghum Grain Mold Disease Complex: Pathogens, Host Responses, and the Bioactive Metabolites at Play

**DOI:** 10.3389/fpls.2021.660171

**Published:** 2021-05-28

**Authors:** Arlyn Ackerman, Anthony Wenndt, Richard Boyles

**Affiliations:** ^1^Cereal Grains Breeding and Genetics, Pee Dee Research and Education Center, Department of Plant & Environmental Sciences, Clemson University, Florence, SC, United States; ^2^Plant Pathology and Plant-Microbe Biology, The School of Integrated Plant Sciences, Cornell University, Ithaca, NY, United States

**Keywords:** sorghum grain mold, mycotoxins, disease resistance, host-pathogen interactions, phenolics, phenylpropanoid pathway, flavonoid pathway

## Abstract

Grain mold is a major concern in sorghum [*Sorghum bicolor* (L.) Moench] production systems, threatening grain quality, safety, and nutritional value as both human food and livestock feed. The crop’s nutritional value, environmental resilience, and economic promise poise sorghum for increased acreage, especially in light of the growing pressures of climate change on global food systems. In order to fully take advantage of this potential, sorghum improvement efforts and production systems must be proactive in managing the sorghum grain mold disease complex, which not only jeopardizes agricultural productivity and profitability, but is also the culprit of harmful mycotoxins that warrant substantial public health concern. The robust scholarly literature from the 1980s to the early 2000s yielded valuable insights and key comprehensive reviews of the grain mold disease complex. Nevertheless, there remains a substantial gap in understanding the complex multi-organismal dynamics that underpin the plant-pathogen interactions involved – a gap that must be filled in order to deliver improved germplasm that is not only capable of withstanding the pressures of climate change, but also wields robust resistance to disease and mycotoxin accumulation. The present review seeks to provide an updated perspective of the sorghum grain mold disease complex, bolstered by recent advances in the understanding of the genetic and the biochemical interactions among the fungal pathogens, their corresponding mycotoxins, and the sorghum host. Critical components of the sorghum grain mold disease complex are summarized in narrative format to consolidate a collection of important concepts: (1) the current state of sorghum grain mold in research and production systems; (2) overview of the individual pathogens that contribute to the grain mold complex; (3) the mycotoxin-producing potential of these pathogens on sorghum and other substrates; and (4) a systems biology approach to the understanding of host responses.

## Introduction

**FIGURE 1:**
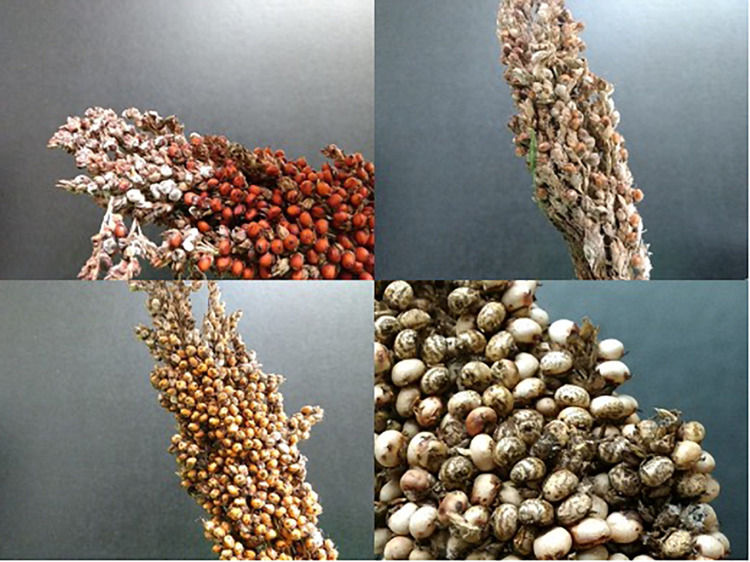
Sorghum grain mold affecting the panicles of tannin and non-tannin cultivars.

Sorghum [*Sorghum bicolor* (L.) Moench] is grown internationally for its robust ability to withstand harsh climates, underlined by great water use efficiency and a diverse biochemical profile boasting high antioxidant potential. Sorghum exhibits robust environmental resilience and yield stability while being capable of reaching potential yield levels at lower input costs than other major cereals. These desirable traits have the crop posed for increased acreage. However, in many areas of agronomic sorghum growth such as regions in the tropical and subtropical climates, high heat and extended periods of humid conditions (relative humidity > 70%) are common. Regions such as these demonstrate inherently warm air capable of holding increased levels of water vaper, and will become more common and expansive as climate change occurs (Willett et al., 2014). Unfortunately, high heat and humidity are conducive to the growth of fungi associated with the sorghum grain mold (SGM) disease complex (Figure 1). Due to the sustained humidity in existing production environments (Western and Southern Africa, Southern Asia, South America, and Eastern North America), grain mold unfortunately persists throughout the growing season and post-harvest through the off-season if warm and wet storage conditions occur (Tola and Kebede, 2016). Storage conditions conducive to post-harvest mold generally occur when high-moisture grain is not properly dried prior to storage, resulting in storage of wet grain and generally higher levels of non-grain plant residue. Storage facilities that lack proper airflow capacity consequently encourage growth of fungal pathogens such as *Aspergillus* spp. from infected grain and residue (Kange et al., 2015). While SGM remains a threat to yield, it is not consistently the top yield-limiting disease in sorghum as it has been called on occasion in the past. The greater concern in regard to SGM is that the associated mycotoxin contamination is indisputably one of the top international threats to sorghum grain quality and safety. Critical reviews on SGM were published by Castor and Frederiksen (1980), Williams and Rao (1981); Forbes et al. (1992), Bandyopadhyay et al. (2000), and Waniska et al. (2001). However, it has been two decades since an updated review of grain mold literature has been provided, and much progress has been made toward understanding the SGM disease complex from physiological, genetic, and biochemical perspectives.

The SGM pathosystem is exceedingly dynamic, as grain mold is innately multifarious, consisting of a multitude of fungi demonstrating various trophic lifestyles: necrotrophic, saprophytic, and hemibiotrophic. The taxonomic diversity of the SGM complex most commonly encompasses but is not limited to *Fusarium* spp*., Aspergillus* spp*., Curvularia* spp., *Colletotrichum* spp. and, *Alternaria* spp. (Forbes, 1986; Forbes et al., 1992; Bandyopadhyay et al., 2000; Little et al., 2011; Cuevas and Prom, 2020). The various fungal constituents that comprise the hierarchal makeup of the SGM complex drastically fluctuates throughout sorghum growth, harvest, and storage. The physiological changes that occur as the host transitions from vegetative to reproductive stages as well as the shift from the growing season into post-harvest storage environments affect the fungal constitution of the SGM complex (Forbes et al., 1992; Bandyopadhyay et al., 2000). The ascomycete *F. verticillioides*, previously known as *F. moniliforme* (Seifert et al., 2003), is the single most predominant species during the growing season (Williams and Rao, 1981; Cuevas et al., 2019a). *Aspergillus* however, is regarded as a top threat to the storage of grain, flourishing in poor storage conditions with high moisture or the presence of insects (Amaike and Keller, 2011; Gemede and University, 2016).

During early host anthesis (i.e., flowering), fungal constituents of the grain mold complex can infect and colonize spikelet tissue prior to grain development (Forbes et al., 1992), resulting in “blasting” of the grain and poor seed set. Post-anthesis, grain mold can externally colonize intact grain outside of the pericarp; however, true damage arises from internal colonization that can hinder grain fill and be detrimental to grain quality. Internal infection of grain is generally a consequence of opportunistic fungal infection at sites of deterioration due to weathering of developing or mature grain. Consequences of internal infection of grain are: (1) digestion of the starches and proteins within the endosperm; (2) overall softening and decay of the seed; and (3) the excretion of toxic secondary metabolites called mycotoxins into the caryopsis, with the latter having one of the most detrimental effects to overall quality and safety (Rodriguez-Herrera et al., 2000). In brief, exposure to dietary mycotoxins has been associated with health adversities such as cancer and cirrhosis (Wild and Gong, 2010; Ostry et al., 2017), immunological disorders (Corrier, 1991), and impaired child growth indicators (Lombard, 2014). In India, a foodborne diarrheal disease outbreak in the 1990s was linked to the consumption of mycotoxin-contaminated sorghum and maize (*Zea mays*) (Bhat et al., 2008). In addition to human diseases, about 48% of total sorghum grain is used as animal feed (Astoreca et al., 2019). In livestock, economically important pathologies associated with mycotoxins, such as Turkey X disease, and other exposure-associated nutritional deficiencies, are pervasive in global animal production systems (Bhat et al., 2010).

Worldwide, mycotoxin exposure through food is widespread (Astoreca et al., 2019) and the amount of international crops affected by mycotoxins was previously estimated near 25% (Park et al., 1999). Park et al. (1999) cited (Boutrif and Canet, 1998) as supporting evidence – even though no mention of this 25% estimate is present in the 1998 publication. This issue was recognized and addressed by Eskola et al. (2019), concluding that the original estimate of 25% could be based on EU legislation and Codex Alimentarius limits. The authors argue this estimate could largely understate the percentage of total food crops that could be infected with mycotoxins at a detectable level, which is argued by (Eskola et al., 2019) to be upward of 60–80%. Estimates for economic losses as a result of SGM-related damages have been previously estimated at US $130 million globally (Das et al., 2020), however, the United States Department of Agriculture (USDA) Grain Fungal Diseases and Mycotoxin Reference states economic losses due to related mycotoxins can be difficult to accurately assess (Schmale and Munkvold, 2009; USDA-ARS, 2018a, b).

The USDA estimates that 300–400 mycotoxins have been identified to date (USDA, 2016), with a portion of these being notable as they are produced by various pathogens implicated in the SGM complex and adversely affect marketable grain quality to an extent to which they are a severe threat to international food and feed safety (Abrunhosa et al., 2016); aflatoxins, ochratoxins, zearalenone (ZEN), fumonisins, deoxynivalenol (DON) and patulin are commonly listed among this subset (Bandyopadhyay et al., 2000; Bhat et al., 2010; Omotayo et al., 2019). Sorghum is the fifth most-produced cereal globally and a staple food in many parts of the world, such as Africa and Asia. Consequently, sorghum grain is responsible for a considerable portion of the food-related mycotoxin exposure as described above. However, unlike other cereals, sorghum does not yet have legislation regulating the maximum mycotoxin concentration in commercial grain (Astoreca et al., 2019).

## SGM-Associated Fungi and Their Mycotoxin Biosynthetic Potential

The pathogenic relationships between SGM-associated fungi and their sorghum host have been difficult to characterize due to the dynamic spatiotemporal development and multi-organismal nature of the disease complex. SGM has generally been regarded as a single entity, yet the complex does not have a single causal agent. Rather, SGM is a syndrome attributable to a diverse assemblage of fungal taxa (Mpofu and Mclaren, 2014), the composition of which can be highly variable across regions (Table 1). Additionally, the fungal makeup of the complex is heavily influenced by fluctuating dominance among species in the hierarchy throughout the lifespan of the host (Hareesh et al., 2000), which ultimately has a direct effect on the potential for specific production of various mycotoxins. Thus, both pathogen-host and pathogen-pathogen interactions are important determinants of SGM disease outcomes, further convoluting prospects for developing stable host resistance.

**TABLE 1:** Summary of SGM-associated fungi implicated in biosynthesis of some major mycotoxins.

**Mycotoxin**	**SGM-associated Fungi**	**Region**	**References**
Aflatoxin	*Aspergillus flavus, A. parasiticus*	Egypt	Abdel-Sater et al., 2017
		Tunisia	Lahouar et al., 2016
		India	Mukherjee and Lakshminarasimham, 1995
Deoxynivalenol	*Fusarium graminearum*	Australia	Blaney and Dodman, 1988
		India	Ramakrishna et al., 1989
Fumonisin	*Fusarium* Section *Liseola*	United States	Leslie et al., 1996
		Italy	Moretti et al., 1995
		Spain	Visconti and Doko, 1994
		Nigeria	Vismer et al., 2019
		Philippines	Leslie et al., 1996
		Burundi	Munimbazi and Bullerman, 1996
		India	Sharma et al., 2011
		Australia	Nelson et al., 1992
Moniliformin	*Fusarium* Section *Liseola*	Argentina	Pena et al., 2019
		United States	Leslie et al., 1996
		South Africa	Leslie et al., 1996
		Philippines	Leslie et al., 1996
Nivalenol	*Fusarium graminearum*	Australia	Blaney and Dodman, 1988
Ochratoxin A	*Aspergillus* Section *Circumdati, Penicillium* spp.	South Africa	Gil-Serna et al., 2011
		Tunisia	Lahouar et al., 2017
Zearalenone	*Fusarium graminearum, F. semitectum, F. incarnatum*	Australia	Blaney and Dodman, 1988
		India	Gupta, 1998
		Japan	Aoyama et al., 2014
		Tunisia	Lahouar et al., 2017

*References and regions correspond to studies confirming mycotoxin biosynthetic ability of sorghum-associated fungal isolates *in vitro*.*

The diverse fungal species associated with SGM each take on unique physical and biochemical relationships with their host, involving both pathogenic and saprophytic lifestyles. Given the breadth and complexity of these relationships, host molecular defense mechanisms and the underlying genetics have been difficult to characterize. The need to breakdown the multi-species complex and scrutinize its various components has been expressed in previous literature (Das et al., 2011; Mpofu and Mclaren, 2014), and numerous efforts have been made in recent years (Cuevas et al., 2019a; Nida et al., 2019; Prom et al., 2020a). However, substantial gaps remain in understanding these relationships, reflected by lack of deployed SGM resistance in sorghum grown in agricultural environments around the world. Additionally, little is known about the ecology and mycotoxin biosynthetic potential of SGM-associated fungi, further limiting the extent to which conclusions can be drawn about the risk of toxin exposure within and across environments. The recent successful efforts in breaking down the components of the disease complex, and understanding pathogenicity on sorghum hosts, have created an opportunity to more critically examine the roles of the implicated fungal taxa in the disease complex and their implications for the future of research regarding SGM.

### *Fusarium* spp.

A predominant genus of SGM-associated fungi is *Fusarium*, which contains numerous mycotoxigenic species and is among the most common genera in the disease complex (Williams and Rao, 1981). The most common species associated with sorghum grain mold are *F. verticillioides* (synonymous with *F. moniliforme* in the literature for the purposes of this review), *F. thapsinum*, and *F. proliferatum*, although many others have also been isolated from sorghum grain (Pena et al., 2019). *F. verticillioides* and related taxa produce the mycotoxin fumonisin, which has been implicated in human and animal diseases such as esophageal cancer, equine leukoencephalomalacia, and impaired growth (Chen et al., 2018). In more temperate growing environments, DON, a mycotoxin produced by *F. graminearum*, is a major concern across susceptible cereal crops (Das et al., 2011; Dweba et al., 2017). Sorghum contamination with DON has been occasionally reported (Das et al., 2011), but *F. graminearum* is demonstrably less virulent in the disease complex than other fusaria, such as *F. thapsinum* (Mpofu and Mclaren, 2014).

#### Mycotoxigenic Characteristics of Sorghum-Associated *Fusaria*

Relatively little is known about the mycotoxin biosynthetic potential of *Fusarium* isolates derived specifically from sorghum. Among isolates of *Fusarium verticillioides* derived from sorghum, much variability has been documented in fumonisin B1 (FB1) biosynthetic potential. Nelson et al. (1991) showed that among 15 sorghum- or millet-derived *F. moniliforme* isolates from sub-Saharan Africa, 6 produced detectable FB1 *in vitro* with a range of 95–2448 μg/g when incubated at 25°C for 31 days (Nelson et al., 1991). In South Africa, by contrast, it has been shown that *F. napiforme* isolates associated with sorghum molds produce very little to no FB1 (Nelson et al., 1992). In Kansas, USA, FB1-producing *Fusarium* isolates produced between 3 and 3148 μg/g FB1 when cultured on cracked maize under the same conditions (Leslie, 1992).

Moniliformin (MON), another mycotoxin produced by *Fusarium* species associated with SGM, can also accumulate in sorghum and be cause for human and animal health concern. In Argentina, sorghum-derived *F. thapsinum* isolates produced 175–2,100 μg/kg MON (mean 579 μg/kg) on sorghum medium *in vitro* when incubated at 28°C for 21 days (Pena et al., 2019). Leslie et al. (1996) found that sorghum-derived *F. verticillioides* isolates from United States, Philippines, and South Africa were capable of producing MON when incubated on ground maize at 25°C for 21 days. While the isolate with the highest biosynthetic potential (10,345 μg/kg) was collected in South Africa, vast ranges in MON production by *F. verticilliodies* was observed across the isolates of the various geographical regions (Leslie et al., 1996).

Aside from fumonisins and moniliformin, the *Fusarium* genus is a prolific producer of trichothecene mycotoxins such as deoxynivalenol (DON) and zearalenone (ZEN), and others, which have been documented in sorghum. DON biosynthetic potential or sorghum-derived *Fusarium* isolates has also been studied to some depth in a range of environments. In India, isolates cultured in glucose-yeast extract-peptone broth at 28°C for 14 days exhibited relatively low levels (0.01–0.04 μg/g) of DON biosynthesis (Ramakrishna et al., 1989). By contrast, a *F. graminearum* isolate from Queensland, Australia produced 239 μg/g on ground maize substrate incubated at 28°C for 28 days (Blaney and Dodman, 1988). This divergence in toxigenicity across geographies is consistent with earlier evidence that populations of *F. graminearum* are differential in their abilities to produce toxins and cause disease in their plant hosts (Ward et al., 2008). Aoyama et al. (2015) found that Japanese isolates of *F. semitectum* derived from sorghum hosts produce substantial amounts of ZEN on sorghum substrate, ranging from 1,530 to 19,400 μg/g. This is higher than what has been observed among isolates of *F. semitectum* and other *Fusaria* in India, where ZEN production among toxigenic strains ranged from 0.6 to 2.4 μg/g on rice substrate when incubated at 25°C for 20 days (Gupta, 1998).

#### Fumonisin as a Virulence Factor in Host Diseases

*Fusarium verticillioides*, a major contributor to SGM disease outcomes globally, exhibits both biotrophic and necrotrophic modes of infection. With respect to its role in the sorghum grain mold disease complex, *F. verticillioides* infection typically occurs via aerial spores that colonize developing panicles during florescence (Williams and Rao, 1981). There has been debate regarding whether fumonisins act as virulence factors in *Fusarium*-related plant diseases (Munkvold, 2003; Dastjerdi and Karlovsky, 2015). While the infection of *F. verticillioides* and bioactivity of FB1 within the host has not been extensively studied in sorghum, the etiology of infection via FB1 and fumonisins as a whole has garnered attention in other crops such as maize. There is some evidence, largely from seedling diseases, that fumonisin-producing isolates are more virulent than isolates that do not produce fumonisins (Gilchrist, 1998; Abbas et al., 2000; Bacon et al., 2008). Abbas et al. (2000) for example, demonstrated that FB1 is active on contact and exhibits limited translocation via the xylem when able to penetrate through a wound, thus playing an active role in necrotrophic infection. However, there has been little convincing evidence that fumonisins play a role in *Fusarium* virulence in grain diseases (grain molds, ear rots, etc.).

Several studies have been unable to demonstrate a relationship between fumonisin production and disease outcomes. Dastjerdi and Karlovsky (2015) reported from stalk rot of maize that fumonisin-producing and non-producing strains of *F. verticillioides* were shown to colonize stalks at an equal rate and caused similar levels of ear rot. Additionally, both fumonisin producing and non-producing strains were able to create similar levels of maize ear rot. Igarashi et al. (2013) found in Arabidopsis that the successful suppression of FB1-related host cell death caused by host recognition of FB1 is independent of any phytohormonal signaling response, and is actually a pathogen-associated molecular pattern-triggered immunity. In maize treated with *F. verticillioides*, Campos-Bermudez et al. (2013) found that the transcriptional and subsequent metabolic responses of the susceptible variety were far more accentuated than the resistant, suggesting that metabolite synthesis was actually supporting *F. verticillioides* growth and alluded to a more constitutive form of defense in resistant genotypes (Campos-Bermudez et al., 2013). This statement aligns with current literature as FB1 elicits a salicylic acid (SA)-based response in the host – a response usually reserved for responding to biotrophic invasion (Glazebrook, 2005; Verwaaijen et al., 2019). This SA response results in induced apoptosis via hypersensitive reaction (Torre-Hernandez et al., 2010), a successful mode of defense against a biotroph, but counterproductive in the case of necrotrophic and hemibiotrophic infection, ultimately creating dead host tissue that is optimal for necrotic *F. verticillioides* colonization. These studies illustrate the potential relationship (if any) of fumonisin production to *F. verticillioides* infection (Munkvold, 2003).

### *Aspergillus* spp.

The *Aspergillus* genus contains among the most prolific producers of the mycotoxins aflatoxin (AF) and ochratoxin. The *Aspergillus* species common in the SGM disease complex include soil-borne saprophytes that can opportunistically infect the developing caryopsis following events such as insect damage (Pfliegler et al., 2020). The *Aspergilli* commonly found in sorghum grain molds are: *A. flavus*, *A. niger, A. parasiticus, A. fumigatus*, and *A. glaucus* (Little et al., 2011; Gemede and University, 2016). *A. flavus* and *A. parasiticus* are the most notable producers of aflatoxins, and while *A. fumigatus* produces the immunosuppressive mycotoxin gliotoxin to increase virulence to plants, animals and humans, it is not known to produce aflatoxin (Kamei and Watanabe, 2005). The risks associated with aflatoxin exposure are most pronounced in tropical and subtropical environments, where *Aspergillus* spp. proliferate abundantly, and the risk is less in regions with both cooler and drier off seasons (Cotty et al., 1994; Amaike and Keller, 2011). Additionally, growing regions with the inability to utilize modern grain storing technologies, which boast increased aeration and drying of stored grain, are at risk of enhanced fungal growth during storage.

The consumption of aflatoxin-contaminated grain can lead to aflatoxicoses (Marin et al., 2013; Mahato et al., 2019), which are some of the most widespread mold-associated diseases globally (Latgeì, 1999). Aflatoxin exposure can result in cancer, lack of immune suppression, liver damage, and mortality (Bennett and Klich, 2003; Sarma et al., 2017). From a public health view and in regard to the SGM complex, *A. flavus* and its close relative within the *Flavi* section, *A. parasiticus*, are of the highest concern within the *Aspergillus* genus (Sarma et al., 2017). Compared to *A. parasiticus, A. flavus* has a wide host range, and is much more prevalent in the SGM complex across locations (Amaike and Keller, 2011; Little et al., 2011; Gemede and University, 2016; del Palacio and Pan, 2020). Out of the more than 20 known aflatoxins there are four major aflatoxins which are a focus of studies for their abundance in foods and toxicity: B1, B2, G1, G2, and M1 (Iqbal et al., 2015; Kumar et al., 2017). AFB1 is the most toxic of these four, being a potent genotoxic and hepatoxic agent as well as being classified as a group 2A carcinogen by the International Agency for Research on Cancer (IARC) (Khoury et al., 2011). The *A. flavus* S and L strains are prolific producers of aflatoxins B1 and B2, and in addition, the L strains produce aflatoxins G1 and G2 (Amaike and Keller, 2011).

Under *Aspergillus* infection, sorghum has been shown to produce varying levels of antifungal proteins including sormatin, glucanases, and chitinases (Seetharaman et al., 1997; Ratnavathi and Sashidhar, 2004). Ratnavathi and Sashidhar (2004) showed chitinase as a response mechanism to *Aspergillus* from relatively strong positive correlations between chitinase activity and aflatoxin levels for both white-pericarp (*r*^2^ = 0.482) and red-pericarp (*r*^2^ = 0.600) sorghums. The authors added that the lower rate of chitinase activity in red to white-seeded sorghums is likely due to red-seeded sorghums reliance on a constitutively higher presence of polyphenols. Chitinase and glucanase production in an effort to fragment a pathogen cell wall is a common defense mechanism in host plants (de Ferreira and da Monteiro, 2017), and while further studies in sorghum are limited, chitinase production has been shown to be an effective defense response to *Aspergillus* in maize and peanut (Fountain et al., 2015; Hawkins et al., 2015).

In addition to their roles as pathogens in the SGM disease complex, *Aspergilli* also constitute a serious post-harvest spoilage threat for sorghum and other crops when grain is stored under sub-optimal conditions conducive for fungal growth and mycotoxin accumulation. Post-harvest genetic resistance is especially important in ensuring the prevention of aflatoxin infection throughout storage, as *Aspergillus* remains a top threat to storage safety. Sorghum is a staple crop in many developing countries which lack proper storage facilities capable of limiting contamination, and post-harvest genetic resistance is an important defense measure which prevents further expenses such as fumigation from occurring (Gemede and University, 2016; Meseka et al., 2018; Soni et al., 2020).

Compared to the *Aspergilli* associated with maize and groundnuts, the crops most vulnerable to aflatoxin accumulation in many environments, there is relatively little understanding of the mycotoxigenic characteristics of sorghum-associated *Aspergillus* strains. In parts of the world where sorghum is a major staple, notably India and sub-Saharan Africa, some evidence has accrued enabling characterization of aflatoxin biosynthetic potential of sorghum-derived isolates. In Egypt, Abdel-Sater et al. (2017) found that local sorghum-derived isolates of *Aspergillus flavus* produced an average of 254 μg/kg AFB1 when incubated on potato dextrose agar at 28°C for 10 days. Tunisian sorghum-derived isolates, on the other hand, produced just 1.15 μg/kg AFB1 on whole sorghum grains incubated at 37°C for 7 days (Lahouar et al., 2016). This discrepancy is consistent with earlier evidence that the thermal optimum for aflatoxin production is ∼30°C (Mousa et al., 2011), but could also be indicative of differential mycotoxigenicity of fungal isolates even from the same geographic region.

In India, *Aspergillus flavus* isolates from sorghum grain produced on average 2567 μg/kg AFB1 in yeast extract broth when incubated at 27°C for 7 days (Usha et al., 1994). Other studies in India also report aflatoxin biosynthetic potential in this ballpark in culture media (Somashekar et al., 2004; Reddy et al., 2011). Incubation on sorghum grain as a substrate has yielded much lower levels of aflatoxin production by sorghum-derived *Aspergillus* isolates in India (Mukherjee and Lakshminarasimham, 1995). This is illustrative of the importance of substrate characteristics in aflatoxigenesis; Winn and Lane (1978), for example, observed marked differences in aflatoxin deposition across sorghum substrates in different forms (whole, cracked, and ground) (Winn and Lane, 1978).

### *Curvularia* spp.

Another ascomycete genus commonly associated with SGM is *Curvularia*, particularly *C. lunata*, which has been shown to play an increased role in the disease complex in drier environments and to have greater negative impact on germination in early season (Prom et al., 2016). Similarly, to *F. verticillioides*, *Curvularia* is typically associated with early-stage infection of the developing sorghum caryopsis and is known to elicit defense response genes in the host plant (Little and Magill, 2003). There is some evidence that *Curvularia* may be more competitive in the disease complex than *Fusarium* spp. in moist environments and that infection by this fungus substantially reduces seed viability even without presenting severe symptoms of infection (Prom et al., 2003). While certainly regarded as an important disease agent in SGM, there is no confirmed evidence of mycotoxin deposition by *Curvularia* in sorghum and it is unlikely that this genus plays and active role in sorghum mycotoxin contamination.

### *Colletotrichum* spp.

The *Colletotrichum* genus, also the causal agent of another economically important sorghum disease, anthracnose, is known to play a role in the SGM disease complex. The predominant species implicated in both pathosystems is *C. graminicola*, which is abundant in warm, humid sorghum production contexts and has high levels of genetic diversity (Cuevas et al., 2016). While *Colletotrichum* spp. can occasionally predominate over other taxa in the SGM disease complex (Bandyopadhyay et al., 2000), the relative importance of this species is likely highly dependent on climatic conditions in the growing environment. In Texas, a low-humidity environment, for example, there is evidence that *Colletotrichum* spp. is a far less prominent member of the fungal assemblage than *Fusarium, Curvularia*, or *Alternaria* taxa (Prom et al., 2011). Like *Curvularia* spp., *Colletotrichum* is likely not implicated directly in mycotoxigenesis in the SGM disease complex. However, it remains unknown the extent to which atoxigenic taxa involved in SGM symptom manifestation modulate or enable toxin deposition by toxigenic fungi via disruption of physical, biochemical, or immune defenses of the host – this should be the subject of future investigation into the host-pathogen and pathogen-pathogen interactions within the disease complex.

### *Alternaria* spp. and *Epicoccum sorghinum*

While not known to infect early flowering tissue of sorghum, *A. alternata* and *A. solani* are common contributors to SGM disease symptoms and have also been implicated in mycotoxin contamination (Bandyopadhyay et al., 2000). In certain environments, such as Texas and Turkey, this genus can be the most prominent member of the SGM fungal assemblage (Prom et al., 2020a). *Alternaria* is a diverse genus of ascomycete fungi, which is a prolific mycotoxin producer and is implicated in a range of plant diseases, notably in high-value crops (Logrieco et al., 2009). Like *Fusarium* spp., the mode of infection of *Alternaria* spp. in the SGM disease complex is largely via airborne spores, which are present in the growing environment throughout caryopsis development (Bandyopadhyay et al., 1991). Sorghum-associated *Alternaria* can be prolific producers of key mycotoxins such as tenuazonic acid, alternariol, alternariol methyl, altenuene, and altertoxin. The aforementioned *Alternaria* mycotoxins can be mutagenic and cytotoxic and, and while less toxic than aflatoxins or fumonisins, are believed to have synergistic effects (Patriarca et al., 2007). At present, tenzuazonic acid contamination in sorghum- or millet-based infant foods is the only governmentally regulated *Alternaria* toxin (Tralamazza et al., 2018).

Sorghum contamination with *Alternaria* mycotoxins has been observed in many production contexts globally. Despite the known role of this genus in the disease complex, relatively little attention has been given to the toxigenicity of *Alternaria* associated with SGM, compared to higher-profile toxins in public health dialog. In India, isolates of sorghum-derived *Alternaria tenuissima* produced 32.6, 22.1, 9.2, 2.7, and 1.8 μg/g of these toxins, respectively, on rice medium incubated at 25°C for 28 days (Bilgrami et al., 1995). *Epicoccum sorghinum* (formerly *Phoma sorghinum*) is another important contributor to SGM symptoms in some production systems and has also been implicated in the production of tenuazonic acid. A recent study from Brazil documented substantial tenuazonic acid production by sorghum-derived isolates between 0.0986 and 148 μg/g incubated on rice substrate at 25°C for 21 days (Oliveira et al., 2019).

## Mechanisms of Grain Mold Resistance in Sorghum

Previous publications dating from the 1980s to the early 2000s have described important sources of host resistance to sorghum grain mold, recognized and selected upon through visual features such as panicle compactness (Williams and Rao, 1981; Brown et al., 2006), grain pigmentation representative of tannin content, presence of a pigmented testa, grain hardness, endosperm texture, pericarp thickness, and both glume coverage and tenacity (Williams and Rao, 1981; Menkir et al., 1996; Brown et al., 2006; Sharma et al., 2010). A study by Sharma et al. (2010) using the sorghum mini core collection (Upadhyaya et al., 2009) found two major phenotypes to contribute to SGM resistance in significant ways: panicle compactness and grain pigmentation. Panicle structures exhibiting compactness were found to significantly increase grain mold severity (*r* = 0.47) and grain pigmentation to decrease severity (*r* = −0.45). It should be noted that the relationship between grain pigmentation and SGM is difficult to determine using visual methods due to potential bias, as it is inherently easier to observe mold incidence on white pericarp grain than pigmented grain.

Open panicle structures common in the guinea race of sorghum allow increased resistance to mold colonization by maximizing airflow and minimizing moisture within the panicle. Additionally, guinea sorghum exhibits large glumes with high grain coverage, a trait shown to limit grain mold infestation (Audilakshmi et al., 1999). However, guinea sorghum commonly demonstrates poor yield as a result of less grain per branch than other sorghum races and exhibit undesirable agronomic traits. Consequently, most commercial varieties consist of semi-open to semi-compact panicle architecture rather than open. This trend demonstrates the limitations of relying on open-panicle structure for SGM resistance.

While panicle architecture is a compilation of types of inflorescence branching, grain pigmentation is a culmination of a diverse and abundant set of biochemical compounds housed within the pericarp, testa, and endosperm (Waniska, 2000). Presence of a dominant spreader gene is necessary for the spread of pigmentation from the testa into the pericarp (Xiong et al., 2019b). Glume pigmentation is also influenced by biochemical compounds similar to the grain. A large portion the biochemical compounds that influence grain and glume color consists of the secondary metabolites that fall into the chemical class of phenolics (Shen et al., 2018), many of which have demonstrated biochemical host resistances that are active against SGM infection. Ubiquitous throughout plants, phenolics of sorghum are responsible for reacting to environmental cues and responding to stress (Cheynier, 2012; Razzaq et al., 2019). Total phenolics in sorghum grain have previously been broken down into phenolic acids, condensed tannins, and flavonoids, all of which have been implicated into SGM resistance at some level (Shen et al., 2018; Xiong et al., 2019b).

Phenolic compounds underlying pigmentation in sorghum demonstrate a range of antioxidant potential that provides an effective source of phytochemical resistance to diseases such as SGM through scavenging of reactive oxygen species generated during fungal infection by both the host and pathogen (Das and Roychoudhury, 2014). While constitutive maintenance of phenolic compounds is widely present in sorghum grain, synthesis of critical phenolic compounds with increased specificity to biotic resistance is largely induced in response to pathogen detection (Liu et al., 2010). Due to this responsiveness of inducible phytochemical production, total phenolic content in sorghum grain unchallenged by pathogens does not effectively predict biotic resistance (Dicko et al., 2005; Atanasova-Penichon et al., 2016).

The presence of a pigmented testa contributes a large amount of condensed tannins and a considerable portion of phenolic content to the biochemical profile of sorghum grain (Menkir et al., 1996). However, the use of condensed tannins within a pigmented testa as a primary source of SGM resistance is limited as tannins lower feed acceptance (Rooney and Murty, 1982) and hinder protein digestion in both animals and humans (Wu et al., 2012), as well as reduce the efficiency of grain starch to ethanol conversion (Zhao et al., 2009). Forbes et al. (1992) stated the need to understand resistance mechanisms of varieties without a pigmented testa (no to low condensed tannins), and since the publishing of this review, progress has been made in elucidating the grain mold – sorghum pathosystem and understanding the host phenolic profile far beyond tannin content. Important sources of both inducible and constitutive phytochemical resistances have been further characterized; largely enhanced by an improved understanding of the underlying metabolic pathways and processes in sorghum.

## Bioactive Metabolites and Pathways Involved in Host Response to Grain Mold Infection

Improved knowledge of the transcriptional responses and metabolic architecture of not only sorghum, but crops such as wheat (*Triticum aestivum*) and maize (that encounter similar head mold diseases), has guided the characterization of cereal grains response to grain mold-specific pathogens to limit host stress and mitigate mycotoxins production. Increased characterization of the head mold pathosystems has provided a framework to further understand host phytochemical resistances of sorghum that are derived from metabolic pathways. The following sections seek to highlight specific host chemical mechanisms that are active throughout SGM disease development to provide a general understanding of these interactions to assist in the further research and development of sorghum cultivars with effective phytochemical grain mold resistances while maintaining and even improving important agronomic traits.

In sorghum, grain pigmentation is underlined by a diverse metabolic profile, and has been used to visibly select resistance-related phytochemical traits. Many of these chemical compounds underlying grain pigmentations are phenolic compounds, which represent a large group of secondary metabolites present across the plant kingdom that are synthesized in response to biotic and abiotic stress (Bhattacharya et al., 2010). Various phenolic compounds that play roles in SGM resistance are individual products and intermediaries of the phenylpropanoid biosynthesis pathway. Induced biosynthesis of these phenolics as a defense mechanism occurs predominantly in resistant sorghum genotypes (Katilé et al., 2010; Prom et al., 2020a).

### The Phenylpropanoid Biosynthesis Pathway

**FIGURE 2:**
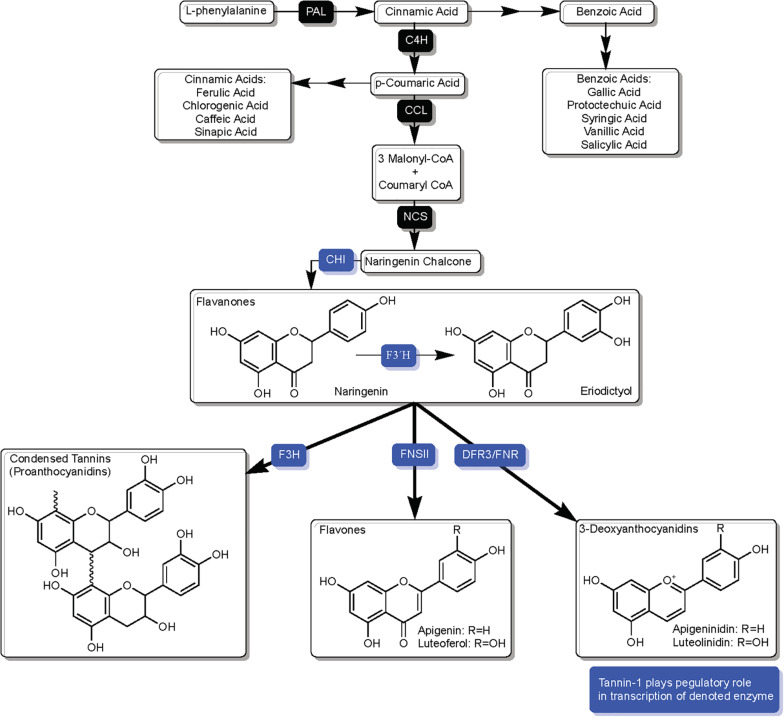
Overview of phenylpropanoid pathway in sorghum, highlighting components and branches which are directly related to SGM resistance/susceptibility and enzymatic competition over flavanones naringenin and eriodictyol. Solid black squares over arrows represent enzymes and blue squares over arrows represent enzymes that are affected by TAN1’s regulatory role in transcription. Enzyme names are as follows: phenylalanine ammonia lysase (PAL), cinnamate-4-hydroxylase (C4H), coumaryl-CoA ligase (CCL), naringenin chalcone synthase (NCS), chalcone isomerase (CHI), flavonoid 3′ hydroxylase (F3′H), dihydroflavonol reductase (DFR), and DFR-like flavanone 4-reductase (FNR).

The phenylpropanoid pathway begins with the enzymatic conversion of phenylalanine by phenylalanine ammonia lysase (PAL) (Tohge et al., 2013), which then undergoes additional enzymatic conversions resulting in naringenin chalcone, the intermediate that chalcone isomerase (CHI) ultimately converts to the flavanone naringenin and marks the beginning of the flavonoid biosynthesis pathway (Figure 2). Many of the phenolic acids contributing to SGM disease resistance are derivatives of phenylalanine, and while part of the phenylpropanoid pathway, are not part of the downstream flavonoid pathway.

Phenolic acids represent the largest and simplest group of non-flavonoid phenolics in sorghum grain. Having been subject to an array of anti-mycotoxin research, phenolic acids have been shown to both inhibit and activate mycotoxin production (Atanasova-Penichon et al., 2016; Stuper-Szablewska and Perkowski, 2017). The main phenolic acids connected to SGM resistance reported in sorghum consist of the (1) cinnamic acids: ferulic, chlorogenic, caffeic, p-coumaric, and sinapic acids (2) benzoic acids: gallic, protocatechuic, vanillic, and syringic acids (Xiong et al., 2019b). In cereals, cinnamic acids such as ferulic and *p-*coumaric acid have been shown to have inhibitory effects for *Fusarium* growth and mycotoxin production (Ferruz et al., 2016b), and additionally shown to directly inhibit *F. verticillioides* and *F. proliferatum* mycelial proliferation and fumonisin production on maize-based media (Ferrochio et al., 2013). Studies have shown caffeic acid and vanillic acid to drastically reduce FB1 production and mycelial growth of *Fusarium* (Beekrum et al., 2003; Schöneberg et al., 2018). In contrast to cinnamic acids, benzoic acids with the exception of syringic acid have generally been shown to have activating effects, even providing slight stimulation to mycelial growth (Boutigny et al., 2009; Atanasova-Penichon et al., 2016; Schöneberg et al., 2018).

Phenolic acids exist in both bound and free forms. Bound forms represent 70–95% of phenolic acid in sorghum grain (Xiong et al., 2019b) and present a multifaceted grain mold resistance source. Alongside of contributing to antioxidant potential and limiting mycotoxins on a cellular level, bound forms of phenolic acids strengthen grain hardness (Chiremba et al., 2012). Alongside ferulic acid, *p-*coumaric acid has also been shown to positively correlate with sorghum grain hardness (Chiremba et al., 2012). Grain hardiness contributes to grain mold resistance by reducing weathering of the pericarp and ultimately limits the ability of grain mold pathogens to degrade the endosperm. This connection of phenolic acids to grain hardness suggests an interesting contribution to SGM-resistance (Jambunathan et al., 1992; Waniska et al., 2001).

Compared to bound phenolic acids, free phenolic acids represent a smaller portion of total phenolic acids within sorghum, maize and wheat. Additionally, biosynthesis levels undergo more drastic fluctuations than their bound counterparts (Atanasova-Penichon et al., 2016). In many cereals, free chlorogenic acid is the most common free phenolic acid. Gauthier et al. (2016) demonstrated in maize that chlorogenic acid is a valuable source of resistance to *F. graminearum* proliferation and limiter of type B trichothecene mycotoxin production. While chlorogenic acid exhibits antifungal properties of its own, the authors showed that *F. graminearum* transforms host chlorogenic acid into caffeic acid, which exhibits antifungal properties of increased potency over the former. The authors concluded this phenomenon regarding chlorogenic acid exhibits “pro-drug” qualities, demonstrating its highest toxicity levels when degraded into caffeic acid (Gauthier et al., 2016). Chlorogenic acid is shown to be a major phenolic acid present in sorghum (Pasha et al., 2015), shown to play roles in essential physiological processes such as photosynthesis (Turner et al., 2016); however, the role of chlorogenic acid as a fungal inhibitor in sorghum has not been explored to the extent that it has in maize or wheat.

Both the strain of pathogen and host are critical determinants in the overall efficiencies of phenolic acids as they interact with pathogens in inhibitory, neutral or activating roles (Ferruz et al., 2016a; Gauthier et al., 2016). Research has helped create a solid understanding of the roles phenolic acids play in diseases such as wheat Fusarium head blight and maize ear rot. Even though sorghum boasts a diverse and abundant phenolic profile well suited for further exploration in relation to biotic resistance, phenolic acid activity has not been as extensively studied with sorghum-based substrates using grain mold pathogens and related mycotoxins. Presence of common phenolic acids across crops and studies showing functional properties of phenolic acids on mycotoxins demonstrates a potential for increased contribution to grain mold resistance in sorghum. To understand the nuances of phenolic acid based-sorghum grain mold resistances, interactions of phenolic acids with grain mold pathogens must undergo further research using sorghum-based media or *in vivo* studies to understand these relationships *in planta*.

### The Flavonoid Biosynthesis Pathway: Naringenin as a Precursor Influenced by Host Sensitivity

Naringenin marks the beginning of the flavonoid biosynthesis pathway, acting as a substrate that is subject to competition from an array of enzymes: flavonoid 3′ hydroxylase (F3′H), flavanone-3-hydroxylase (F3H) as well as dihydroflavonol 4-reductase (DFR) and the DFR-like enzyme flavanone 4-reductase (FNR) (Xiong et al., 2019a; Figure 2). The ultimate fate of naringenin is heavily influenced by genotype by environment interactions, with studies such as (Taleon et al., 2012) demonstrating 48% of total variation in naringenin abundance being a consequence of environmental variation, and separate studies having reported major influence on naringenin by biotic factors such as fungal ingression (Boddu et al., 2005). The fate of naringenin is also tissue-dependent, as downstream products such as 3-deoxyanthocyanidins (3-DAs) are maintained constitutively in unchallenged grain while synthesis is only induced in leaf tissue upon fungal ingress (Boddu et al., 2005; Ibraheem et al., 2015; Kawahigashi et al., 2016).

Sorghum demonstrates a high level of host sensitivity to fungal ingression, as spatial accuracy in the induction of phytoalexins critical to SGM resistance occur through site-specific synthesis at place of infection. Naringenin is a precursor to many of these phytoalexins and marks an important junction in the flavonoid pathway. A resistant response to fungal ingression is dependent on the availability of naringenin to enzymes that synthesize SGM resistance-related compounds. As described in detail in later sections, synthesis of 3-DA phytoalexins is an inducible resistance-related host response to SGM, and a lack of activity by F3H can be a consequence of inducible synthesis of 3-DAs resulting from increased channeling of DFR3/FNR on naringenin (Figure 2; Liu et al., 2010). As will be explored throughout this text, the availability of naringenin at optimal time and point of infection is crucial to host response. At its core, a large portion of host biochemical responses to grain mold may in many ways a consequence of the management of the enzymatic competition over naringenin.

### Enzymatic Conversion of Naringenin by F3H – The Synthesis and Properties of Condensed Tannins (Proanthocyanidins)

Condensed tannins (proanthocyanidins) are well understood for their roles in increased antioxidant capabilities and significant correlations with SGM resistance (Harris and Burns, 1973; Forbes, 1986; Melake-Berhan et al., 1996; Dicko et al., 2005; Cuevas et al., 2019b). The presence of condensed tannins throughout SGM resistant germplasm is interesting, as there is little knowledge to suggest condensed tannins demonstrate direct toxicity to fungal pathogens as do products of other branches of the flavonoid biosynthesis pathway such as 3-DAs and flavones (Melake-Berhan et al., 1996; Du et al., 2009; Nida et al., 2021). Regardless, the use of condensed tannins is limited in sorghum germplasms for their reduction in protein digestibility of animals. However, the toxicity of condensed tannins to humans has been misunderstood, and have been shown to provide antioxidants, fiber and reduce obesity as a food source (Dykes et al., 2005). Sorghum tannin types have been divided amongst three categories: type I) no tannins; type II) tannins in pigmented testa; type III) tannins in pigmented testa and pericarp (presence of spreader gene) (Waniska et al., 2001).

**FIGURE 3:**
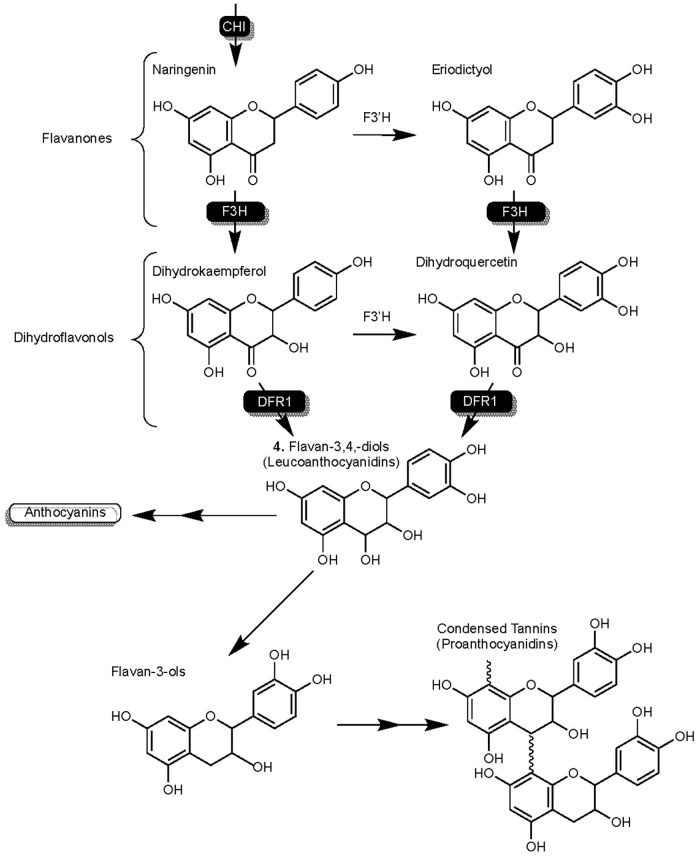
Detailed overview of the flavonoid biosynthesis branch responsible for the synthesis of condensed tannins (proanthocyanidins) in sorghum. Highlights F3H activity on flavanones naringenin and eriodyctiol. Enzymes are as follows: chalcone isomerase (CHI), flavanone-3-hydroxylase (F3H), and dihydroflavonol reductase (DFR).

*SbF3H1* of sorghum codes the flavanone-3-hydroxylase (SbF3H) enzyme that is capable of converting naringenin to condensed tannins (Figure 3). As another competitive enzyme for naringenin, host management of SbF3H activity could play an important role in managing enzymatic competition for naringenin (Mizuno et al., 2014). SbF3H converts flavanones to dihydroflavonols from which SbDFR1 synthesizes flavan-3,4,-diols (leucoanthocyanidins). Leucoanthocyanidins represents a junction in this branch of flavonoid synthesis from which anthocyanidins or flavan-3-ols can be synthesized. Condensed tannins are the result of oligomerization and polymerization of flavan-3-ol compounds (He et al., 2008; Wu et al., 2012).

Early understandings of SGM resistances recognized condensed tannin content as a lone dominant influence on SGM resistance (Harris and Burns, 1973). While this outlook was justified at the time, it was chemical studies such as Menkir et al. (1996) that began to expand this perspective to consider a more complete host phenolic profile by showing inconsistencies in condensed tannin content of SGM resistant phenotypes. Recent studies have assisted in the accurate understanding of the contributions of condensed tannin content to SGM resistance as the expansive genetic underpinnings of grain mold resistance continue to be elucidated. Biosynthesis of condensed tannins is controlled by the *Tannin-1* (*TAN1*) transcription factor (Wu et al., 2012). *TAN1* demonstrates influence over enzymatic competition for naringenin by regulating expression of enzyme-coding genes in the pericarp responsible for the transcription of *CHI, F3H, F3′H, DFR* and *ANS* (Wu et al., 2012; Mizuno et al., 2014), and consequently influencing the synthesis of 3-DAs, flavones, and anthocyanins in addition to condensed tannins (Liu et al., 2010).

Wu et al. (2012) found the *TAN1* allele was present throughout 78% of a diverse collection of sorghum germplasm consisting of 161 accessions. Similar to the 161 accessions used by Wu et al. (2012), Cuevas et al. (2019a) found that a functional *TAN1* is present in 79% of the sorghum association panel (SAP) (Casa et al., 2008), an interesting finding considering that a majority of the accessions within the SAP were also found be highly susceptible to SGM. The study by Cuevas et al. (2019a) demonstrated that genotypes harboring a non-functional *TAN1* allele (*tan1-a)* were more susceptible to grain mold on average compared to accessions with the functional allele. Interestingly, the Genome-Wide Association Studies (GWAS) of the SAP did not associate *TAN1* with grain mold resistance, illustrating the complexity of the SGM resistance phenotype that supports the author’s conclusion that resistance is a culmination of many additional factors in addition to the concentration of tannins (Cuevas et al., 2019a).

Another recent SGM association study employing 635 Ethiopian genotypes by Nida et al. (2021) identified a collection of protein-coding loci, and consistently detected SNPs within the *TAN1* coding region. The authors suggested *TAN1’*s role in the flavonoid pathway affecting biosynthesis of 3-DAs and tannins could contribute to *TAN1’s* role in grain mold resistance. While widespread presence of *TAN1* across mapping populations is a factor of both aforementioned studies, a key difference is the differing levels of resistance between the two populations, with the former (SAP) demonstrating increased susceptibility overall than the latter (Ethiopian). This notable difference in SGM resistance between mapping populations may be a consequence of varying levels of *TAN1’s* role in transcriptional regulation of the enzyme-coding genes in the flavonoid pathway, and could be factoring into why *TAN1* was not associated with SGM resistance in the SAP, but consistently identified in the Ethiopian panel.

Additional GWAS such as those by Prom et al. (2020a) and Ahn et al. (2019) further highlight the genetic complexities of SGM resistance, as many identified genes show minor rather than major effects, such as a variety of zinc finger proteins, resistance genes (R-genes), and the underlying genetics associated with systemic acquired resistance (SAR) mechanisms (Ahn et al., 2019; Prom et al., 2020a). While condensed tannin content remains an important component of host resistance, it only represents a portion of the phenolic profile that contributes to a complex host resistance mechanism. Many additional components of host resistance such as sorghum SAR mechanisms, R-genes and the phenolic profile in its entirety should be explored in order to provide a more complete understanding of mold resistance in sorghum germplasms (Cuevas et al., 2019a). Additionally, at the core of the grain mold resistance phenotype in sorghum is host management of enzymatic competition over naringenin and the ability to ensure naringenin availability to the correct branch of the flavonoid pathway at time of grain mold infection.

### Enzymatic Conversion of Naringenin by SBDFR3/FNR – The Synthesis and Properties of Flavan-4-OLS, 3-Deoxyanothcyanidins, and Phlobaphenes

#### Flavan-4-ols

**FIGURE 4:**
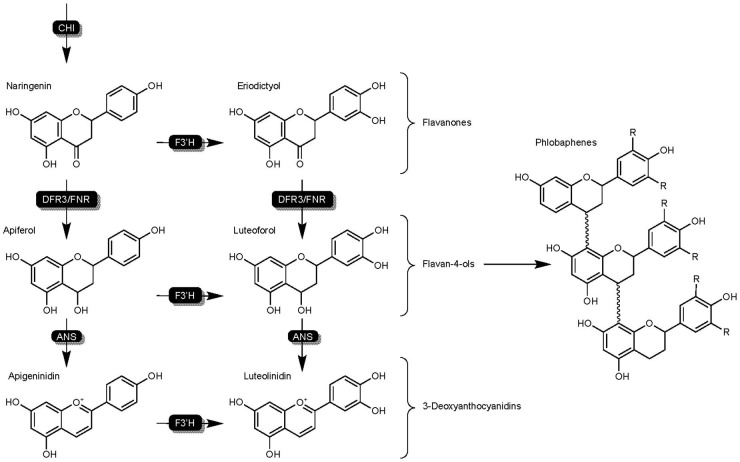
Detailed overview of the flavonoid biosynthesis branch responsible for the synthesis of flavan-4-ols, 3-DAs, and phlobaphenes in sorghum. Highlights DFR3/FNR activity on flavanones naringenin and eriodictyol as well as flavan-4-ols as an intermediate junction between 3-DAs and phlobaphenes. Enzymes are as follows chalcone isomerase (CHI), dihydroflavonol reductase (DFR), DFR-like flavanone 4-reductase (FNR), uncharacterized anthocyanidin synthase (ANS), flavonoid 3′ hydroxylase (F3′H).

Flavan-4-ols have been subject to many early studies regarding grain mold resistance, and repeatedly shown to be a critical component of sorghum defense mechanisms active against SGM (Jambunathan and Kherdekar, 1991; Melake-Berhan et al., 1996). Studies have shown resistant genotypes respond to pathogen infection by producing flavan-4-ols, and demonstrated their importance as a precursor and active compound (Jambunathan and Kherdekar, 1991; Poloni et al., 2014). The dihydroflavonol 4-reductase enzyme SbDFR3 and DFR-like flavonoid 3′-hydroxylases (SbFNR) are responsible for the conversion of naringenin into flavan-4-ol (Kawahigashi et al., 2016; Mizuno et al., 2016) (Figure 4). Resistant and susceptible genotypes have been shown to maintain flavan-4-ols at similar concentrations in early sorghum development (Jambunathan and Kherdekar, 1991). Three weeks post-anthesis however, flavan-4-ol content in susceptible genotypes decreased over three times more than in resistant lines (Melake-Berhan et al., 1996). While flavan-4-ol concentrations have been shown to be present in the caryopsis throughout grain mold resistant genotypes, flavan-4-ols do not directly inhibit the growth of fungal pathogens (Schutt and Netzly, 1991), suggesting a role as a precursor or in signaling mechanisms.

#### 3-Deoxyanthocyanidins

3-DA’s of sorghum are composed of luteolinidin, apigenidin and their methoxylated derivatives (Xiong et al., 2019a). These anthocyanins demonstrate high levels of fungal toxicity and have been tied to fungal resistance mechanisms (Nicholson et al., 1987; Schutt and Netzly, 1991). Early studies such as the one by Menkir et al. (1996) found the phytoalexin apigenidin to contribute to grain mold resistance and found no relationship between resistance with luteolinidin and apigenidin combined. Interestingly, notable studies had previously determined luteolinidin, when compared to apigenidin, exhibits higher levels of fungal toxicity to *H. maydis* and *C. graminicola*, accumulates at slower rates following infection (Nicholson et al., 1987), and is more consistently present throughout anthracnose resistant varieties (Snyder and Nicholson, 1990). While luteolinidin accumulates at slower rates than apigenidin, literature suggest resistant varieties exhibit increased reliance on this extra enzymatic step involving F3′H to produce luteolinidin, possibly to take advantage of the higher fungal toxicity over apigenidin (Nicholson et al., 1987; Mizuno et al., 2014). Melake-Berhan et al. (1996) were able to demonstrate high correlations of apigenidin and luteolinidin with each other as well as with flavan-4-ols; however, the study was unable to demonstrate a major contribution of luteolinidin and apigenidin to grain mold resistance, even though both compounds demonstrate effective fungal toxicity.

3-DAs have been found to be present in maize and flowers of the Gesneriaceae and Bignoniaceae families, but pathogen-induced rapid production of 3-DAs as phytoalexins is unique to sorghum (Liu et al., 2010; Xiong et al., 2019a). In sorghum, 3-DAs are produced in infected cells as inclusion bodies (Snyder and Nicholson, 1990), and migrate to the site of infection (Liu et al., 2010). This production and migration of phytoalexin 3-DAs is primarily a pathogen-induced mechanism, and undetectable in tissues of unchallenged hosts. The enzymes SbDFR3 and SbFNR are responsible for the conversion of naringenin into flavan-4-ol precursors, and an unknown ANS enzyme converts flavan-4-ols to 3-DAs (Figure 4; Xiong et al., 2019a).

Much of the research to understand the transcriptional controls and site-specific production of 3-DAs in response to fungal ingress have been performed in relation to anthracnose resistance using *Colletotrichum sublineolum* (Ibraheem et al., 2010; Kawahigashi et al., 2016). Consequently, much of the research that has highlighted these phytoalexins for site-specificity in sorghum analyzed 3-DA biosynthesis in leaf and mesocotyl tissue. Sorghum fungal diseases such as anthracnose predominantly initiate in leaf tissue, while the most damaging aspects of grain mold occur in developing reproductive tissue (i.e., grain). Leaf tissue production of 3-DAs is primarily an induced mechanism and only measurable when challenged by fungal pathogens (Kawahigashi et al., 2016). In contrast, SGM resistant genotypes maintain constitutive, detectable levels of 3-DAs within grain that is unchallenged by SGM (Awika et al., 2004; Boddu et al., 2005).

Two overlying concepts in the understandings of 3-DA activity in the SGM disease complex outline two shortcomings in current understandings: (1) Antifungal properties and pathogen-related induction of 3-DAs suggests a considerable role in grain mold resistance, but evidence of significant contributions to phenotype are inconsistent; and (2) the analysis of inducible 3-DA activity in SGM specific-interactions and host tissues (developing reproductive tissue, grain, and glume) compared to other fungal pathogens of sorghum has been lacking. Recently however, with the addition of association and expression studies, Nida et al. (2019) successfully identified and connected genes responsible for 3-DA synthesis to SGM resistance and identified induced expression of flavonoid biosynthesis-related structural genes in the grain and glume. The authors performed GWAS for grain mold scores across a large set of 2,010 accessions, with 1,940 Ethiopian landrace accessions, 1,550 of which were from the Ethiopian Biodiversity Institute. This GWAS successfully identified the previously mapped locus that contains the MYB R2R3 transcription factor gene *YELLOW SEED1* (*Y1*) (Nida et al., 2019), that Boddu et al. (2005) had implicated to play a direct role in synthesis of 3-DAs and phlobaphenes in the leaves, glumes and the seed pericarp of sorghum. Furthermore, in the same GWAS, Nida et al. (2019) identified two additional MYB transcription factor genes. The first of which is *YELLOW SEED2* (*Y2*) as previously described by Boddu et al. (2006), as a tightly linked, potentially nonfunctional pseudogene containing 97% sequence identity to the functional *Y1* (Boddu et al., 2006). The second of which is another MYB R2R3 transcription factor gene that was previously uncharacterized, which the authors designated *YELLOW SEED3* (*Y3*). In addition to mapping efforts by the previously illustrated study, Dicko et al. (2005) found higher presence of 3-DA common across the SGM-resistant genotypes within a panel of fifty sorghum varieties, suggesting roles as phytoalexins and contributions to grain mold resistance (Dicko et al., 2005).

The complimentary expression study by Nida et al. (2019) on grain and glume tissue did show the upstream structural gene *SbDFR3* underwent significantly increased expression in resistant genotypes following inoculation of *Fusarium* spp. and *Alternaria* spp. Alongside of maintaining constitutive levels of 3-DAs, induction of genes such as *SbDFR3* suggest a significant degree of responsiveness is present within the grain and glume. Grain and glume *SbDFR3* expression shows transcriptional response to infection, however *SbDFR3* is responsible for the conversion of flavanones to flavan-4-ols, and the enzyme responsible for the conversion of flavan-4-ols to 3-DAs, while likely a form of ANS, remains unknown (Liu et al., 2010). Flavan-4-ols have been shown to positively correlate with 3-DAs, which is promising for 3-DA grain and glume inductions. However, more research is needed to determine if the degree of responsiveness demonstrated by pathogen-induced 3-DA production in leaf, root, and mesocotyl is reflected in SGM niches such as early reproductive tissue as well as mature grain and glume tissues in response to grain mold-specific pathogens. Each host-pathogen interaction is unique (Agrios, 2005; Balint-Kurti et al., 2016), and while many aspects of inducible 3-DA biosynthesis are likely shared between differing pathogen etiologies such as anthracnose and the SGM, each pathosystem should be regarded as unique and extrapolation of knowledge between diseases should be approached cautiously.

#### Phlobaphenes

The alternate fate of flavan-4-ols are phlobaphenes - insoluble phenolic compounds typically stored in the pericarp that contribute to visible red pigmentation of maize and sorghum (Poloni et al., 2014). While most available literature to date has targeted pigmentation as being the primary role of phlobaphenes in plants, recent research in maize highlights phlobaphenes for antioxidant capabilities and roles in resistance to fungal ingression (Landoni et al., 2020). Landoni et al. (2020) reported a strong correlation between phlobaphene content and pericarp thickness (*R* = 0.9318; *p* = 0.0067) and demonstrated it to decrease fumonisin concentration in the seed. The authors also speculate as to the ability of phlobaphenes to inactivate fungal proteins by the formation of irreversible complexes as part of host detoxification mechanisms. Additionally, the enzymatic machinery which synthesizes phlobaphenes from flavan-4-ols has not yet been elucidated. While showing promise in resistance to *Fusarium* spp. of ear rot in maize, phlobaphenes are mainly regarded as a contributor to grain pigmentation in sorghum, and have not been scrutinized for their contribution to other phenotypes such as biotic resistance.

*Pericarp Color 1* (*P1*) of maize, an ortholog to sorghum *Y1*, has been shown to be functionally similar in the maize flavonoid biosynthesis pathway, with a confirmed role in the accumulation of phlobaphenes (Ibraheem et al., 2015; Landoni et al., 2020). *Y1* of sorghum, as described earlier for its role in 3-DA biosynthesis, is primarily known in sorghum for control of pericarp pigmentation (Brenton et al., 2016). In addition to pigmentation, results from Boddu et al. (2005) demonstrate that a functional *Y1* is also required for biosynthesis of phlobaphenes. Additionally, in comparison to maize, sorghum phlobaphenes are present in both vegetative and reproductive tissues; whereas, maize phlobaphenes are generally relegated to reproductive tissues (Boddu et al., 2006; Ibraheem et al., 2015). While both *Y1* and a high presence of flavan-4-ols are present across much of the SGM-resistant germplasm (Menkir et al., 1996; Nida et al., 2019), much is to be determined as to the competition for flavan-4-ols as a substrate. Much progress has been made in understanding the presence of 3-DAs across SGM-resistant sorghum germplasm (Dicko et al., 2005; Nithya et al., 2013); however, the alternate fate of flavan-4-ols and the presence of phlobaphenes across SGM-resistant germplasm has yet to be characterized to the same degree.

### Enzymatic Conversion of Naringenin by SbFNSII – The Synthesis and Properties of Flavones

**FIGURE 5:**
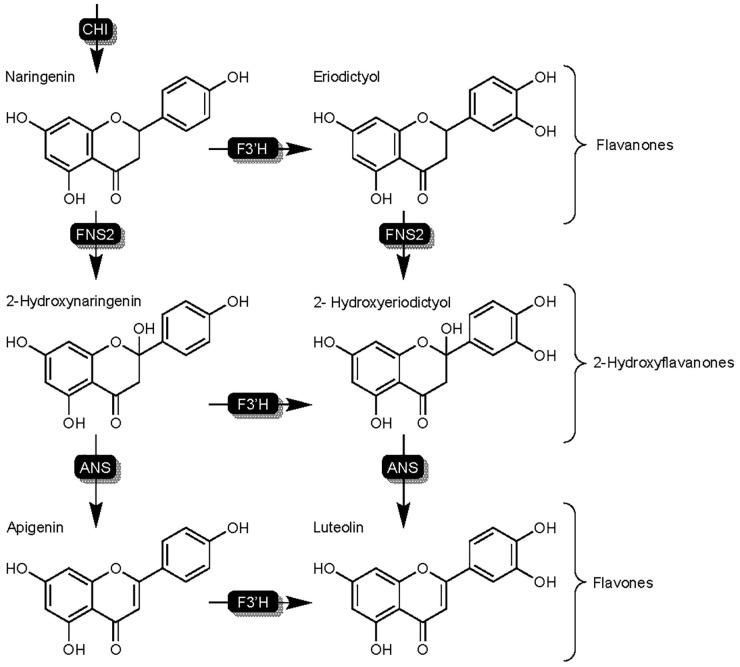
Detailed overview of the flavonoid biosynthesis branch responsible for the synthesis of flavones in sorghum. Highlights FNSII activity on flavanones naringenin and eriodictyol. Enzymes are as follows chalcone isomerase (CHI), flavanone synthase (FNSII).

Flavone synthase II (FNSII) enzymes are responsible for the synthesis of the flavones apigenin and luteolin (Figure 5), and like F3′H, are part of the cytochrome P450 superfamily (Yonekura-Sakakibara et al., 2019). Flavones in sorghum represent a separate branch of the flavonoid biosynthesis pathway that begins with the hydroxylation of naringenin or eriodyctiol by SbFNSII resulting in the formation of 2-hydroxyflavone intermediates (Du et al., 2009). Subsequent conversion of 2-hydroxyflavones by an unknown ANS results in the formation of the flavones apigenin and luteolin (Figure 5). Flavones are one of the largest subgroups of flavonoids and are well understood for their ability to affect color by complexing with anthocyanins as well as antioxidant potential and nutrient value amongst other benefits (Martens and Mithöfer, 2005). Additionally, flavones such as luteolin and apigenin have been shown to demonstrate levels of fungal toxicity of their own, with increased fungal toxicity and higher presence in resistant germplasm demonstrated by the former (Du et al., 2009; Aboody and Mickymaray, 2020). Research in sorghum has suggested flavones are a phytoalexin due to a combination of the aforementioned fungal toxicity and pathogen-related induction (Du et al., 2009). Functional characterization of flavone synthase genes in other crops however, has reported an interesting mechanism in balancing the competition between FNSII and other enzymes and even pathogen-manipulation of flavone production potentially being a component of host susceptibility (Ferreyra et al., 2015).

Darker pericarp color and purple plant sorghum lines have been generally shown to be more resistant to disease (Melake-Berhan et al., 1996; Menkir et al., 1996; Kawahigashi et al., 2016). Kawahigashi et al. (2016) reported that during tissue wounding, red pericarp, purple plant sorghum types favor conversion of naringenin and eriodyctiol by SbFNR (3-DA synthesis), while seemingly suppressing conversion by SbFNSII - suggesting increased reliance on flavan-4-ols and 3-DA phytoalexins pathways over flavones (Dykes et al., 2005; Kawahigashi et al., 2016). On the contrary, tan plants favor conversion of naringenin and eriodyctiol by SbFNSII while seemingly blocking the production of flavan-4-ols, resulting in leaf and glume tissue showing higher levels of apigenin and luteolin over 3-DAs (Kawahigashi et al., 2016; Mizuno et al., 2016). This phenomenon could potentially be a factor contributing to the higher antioxidant capabilities and disease resistance of purple plant/dark pericarp sorghum genotypes over tan plants.

The findings described above from Kawahigashi et al. (2016) suggest potential mechanisms of sorghum in managing enzymatic activity on naringenin to balance compound synthesis between separate flavonoid pathway branches. Research in maize and Arabidopsis has begun to show this as an important trait of SA-mediated resistance, a phytohormone commonly associated with SAR responses. Flavone synthase enzymes responsible for flavone synthesis such as FNSII are widely present throughout the flavonoid biosynthesis pathways of plants. FNSI and FNSII enzymes of maize are functionally similar to SbFNSII, as both play roles in the synthesis of flavones from naringenin and both have been shown to play an important role in the SA – flavone cross talk affecting maize salicylic acid-mediated resistance (Ferreyra et al., 2015; Righini et al., 2019). It is important to note that FNSI distinctly differs from FNSII in that it is part of the 2-oxoglutarate-dependent dioxygenase family and shows higher sequence identity to F3H than to FNSII (Du et al., 2009; Yonekura-Sakakibara et al., 2019). While maize has been shown to rely on both ZmFNSI and ZmFNSII, sorghum has thus far been shown to be reliant on SbFNSII for flavone synthesis (Righini et al., 2019). An important dissimilarity in the roles of FNSI and FNSII exists: FNSI converts flavanones directly to flavones, while most FNSII enzymes convert flavanones to 2-hydroxyflavanones prior to conversion to flavones by an unknown protein (Figure 5; Du et al., 2009; Kawahigashi et al., 2016).

*ZmFNSI-1* is commonly coexpressed with maize R2R3-MYB transcription regulator *P1* (Ferreyra et al., 2015), an ortholog of the critical SGM resistance gene *Y1* (Boddu et al., 2005; Nida et al., 2019). Transcriptional studies show *ZmFNSI-1* is highly active in maize pericarps and silk - tissues commonly under pressure from ear rot-related pathogens (Righini et al., 2019). In maize and Arabidopsis, a SA – flavone cross talk that fine-tunes homeostasis between the phytohormones and flavones has been well described. Studies have characterized an Arabidopsis SA 5-hydroxylase DMR6 (Downy mildew resistance), a recently reported homolog of ZmFNSI-1 (Zhang et al., 2017b). In Arabidopsis, the ZmFNSI-1 homolog DMR6 was shown to be responsible for controlling levels of salicylic acid (SA) and naringenin through use of either compound as a substrate. However, studies show that DMR6 prefers SA as a substrate over naringenin, showing that increased availability of SA over naringenin to DMR6/ZmFNSI-1 will consequently result in higher availability of naringenin. The authors conclude that while enhanced SA levels are responsible for pathogen resistance, lowered levels of apigenin and luteolin are likely not directly connected to resistance. Rather, the resistant mechanism is a result of the preoccupation of ZmFNSI-1/DMR6 with the hydroxylation of SA rather than naringenin, increasing the availability of naringenin as a substrate to other resistance-related enzymes to increase phytoalexin production (Zhang et al., 2017b).

Metabolic studies of sorghum under *Burkholderia andropogonis* (leaf blight) infection point toward induction of flavone synthase as a defense response (Mareya et al., 2019), while studies in Arabidopsis and maize have suggested the potential of flavones in active immune suppression through metabolic manipulation by the pathogen resulting in host susceptibility (Ferreyra et al., 2015). Studies regarding pigmented sorghums suggest a role in limiting SbFNSII activity to support resistance related enzymes such as SbFNR (Dykes et al., 2005; Kawahigashi et al., 2016). Additionally, recent SGM association studies identified BTB/POZ domains within SAR response pathways that involve phytohormone induction (Cuevas et al., 2019a). Rather than SA-induced resistance as described above, Liu et al. (2010) found that expression of *SbDFR3* and 3-DA pathways were induced by methyl jasmonate treatment of sorghum roots and antagonized by SA. The enzymatic competition for naringenin in sorghum and coexpression of *Y1* ortholog *P1* with *ZmFNSI-1* in maize suggest flavones may be carefully managed in SGM interactions; however, host mechanisms must be further characterized in reproductive tissue using grain mold pathogens to elucidate this relationship.

With confirmation of the role of *Y1* in SGM resistance, the combined increased expression of *SbDFR3* and elucidation of implicated SAR mechanisms identified in SGM resistant sorghum suggests there is potential for heightened enzymatic competition for naringenin and a phytohormone-mediated mechanism for management of enzymatic activity on naringenin (Nida et al., 2019; Prom et al., 2020a). The presence of flavones in SGM resistant varieties has been demonstrated, but further functional characterization of SbFNSII in SGM pathosystems is required to understand to which extent that host reliance on flavone synthesis branches of the flavonoid pathway over SbDFR3/SbFNR and F3H-mediated branches contributes to a SGM resistant phenotype.

### Enzymatic Conversion of Naringenin by F3′H – The Synthesis and Properties of Eriodyctiol and 3′ Hydroxylated Derivatives

Part of the cytochrome P450 superfamily, flavonoid 3′-hydroxylase enzyme (F3′H) plays a potentially important role in the SGM response of sorghum as it is responsible for the conversion of naringenin to the flavanone intermediate eriodyctiol (Figure 2; Poloni et al., 2014; Yonekura-Sakakibara et al., 2019). Through hydroxylation of the 3′ position of the B-ring of naringenin, F3′H synthesizes eriodyctiol, the precursor to luteolinidin and luteolin (Figures 4, 5; Mizuno et al., 2016). Luteolinidin has been shown to be present at a higher rate than apigenidin throughout red pericarp sorghum, a pigment trait that correlates with SGM resistance (Dykes et al., 2005). Additionally, luteolinidin exhibited increased fungal toxicity over apigenidin (Nicholson et al., 1987). Luteolin has also been described to exhibit higher presence in disease resistant sorghum lines as well (Du et al., 2009). This phenomenon is suggestive that the extra enzymatic step in F3′H hydroxylation to produce eriodyctiol as an intermediate may play a role in enhancing resistance mechanisms to SGM pathogens.

## Moving Forward With Elucidating SGM Host-Pathogens Interactions and Developing SGM Resistant Cultivars

Research has shown grain mold resistance of sorghum to be a vastly complex and highly quantitative trait (Cuevas et al., 2019a). Recent efforts have begun to isolate the fungal pathogens of the grain mold complex and differentiate host responses. A wealth of knowledge has been provided by recent association studies, but more research is needed to characterize these host-pathogen interactions in both tissue-specific and pathogen-inducible contexts. As more QTL are implicated into SGM resistance, understanding host manipulation of metabolic pathways and pathogen biosynthetic mycotoxin potential is vital to fully elucidating the SGM disease complex and host response mechanisms.

As fungal assemblages change throughout regions, the plasticity of grain mold resistance in current germplasm justifies increased attention. Many studies have been performed over the years screening germplasm in a variety of regions under the stress of different fungal assemblages of the SGM complex (Table 2). As warmer air and widespread humidity becomes more present in the face of climate change, the importance of acknowledging different fungal assemblages in regions with newly developing grain mold pressure will become increasingly important. Much of sorghum grain production relies on hybrid varieties, and considerable positive heterosis for grain mold resistance has been demonstrated by breeding programs (Kumar et al., 2008; Hundekar et al., 2015). Hundekar et al. (2015) reported a better parent heterosis of SGM resistance of 147.22%, but also reported a negative heterotic effect as far as −17.32%. Studies have reported success in developing grain mold resistant hybrid varieties through the use of resistant varieties as the female parent (Thakur et al., 2008), citing the many minor QTL responsible for SGM resistance as a potential factor. While the value of heterosis in SGM resistance has been demonstrated, little is known as to how this phenomenon affects the many biochemical mechanisms described throughout this text. More research is required to better understand the heterotic contributions to the metabolic makeup of sorghum in relation to host resistance.

**TABLE 2:** Table highlighting projects that have screened grain mold resistance in sorghum germplasm at different locations worldwide, under differing fungal assemblages (if listed), and report resistant varieties.

**References**	**Population**	**Number of lines**	**Area of growth**	**Pathogens detected/used if listed**
Bandyopadhyay et al., 1988	World Collection	7,132	ICRISAT, Patancheru, India	N/A
Audilakshmi et al., 1999	Selected lines from NRCS, AICSIP, and ICRISAT	22	Directorate of Oil Seeds Research, Rajendranagar, Hyderabad, India	N/A
Reddy, 2000	ICRISAT - hybrid and parental lines	N/A	ICRISAT, Patancheru, India	N/A
Prom et al., 2003	Selected lines	8	Texas A&M Agricultural Research Farm, College Station, Texas	*F. thapsinum, C. lunata*
Prom, 2004	Selected lines	8	Texas A&M Agricultural Research Farm, College Station, Texas	*Fusarium thapsinum, F. semitectum, Curvularia lunata, Alternaria* spp.
Kumar et al., 2008	ICRISAT - hybrid and parental lines	25	ICRISAT, Patancheru, India	N/A
Thakur et al., 2008	Reevaluation of resistant lines from Bandyopadhyay et al. (1988)	156	ICRISAT, Patancheru, India	N/A
Katilé et al., 2010	Selected lines	12	Texas A&M Agricultural Research Farm, College Station, TX, United States and Greenhouse	*Fusarium thapsinum, Curvularia lunata, Alternaria* spp*., Fusarium semitectum, Aspergillus* spp*., Rhizopus* spp.
Sharma et al., 2010	Sorghum Mini-Core	140	ICRISAT, Patancheru, India	N/A
Prom et al., 2014	US Comercial Hybrids w/breeding line checks	28	Agronomic Research Stations in Bambey and Nioro, Sengal, Africa	N/A
Hundekar et al., 2015	AICSIP - hybrids and parental Lines	44	Sorghum Improvement Project, University of Agricultural Sciences, Dharwad	N/A
Cuevas et al., 2016	Exotic germplasm accessions from Burkina Faso and South Africa	80	Isabela, Puerto Rico	*Fusarium thapsinum, Fusarium semitectum, and Curvularia lunata*
Prom et al., 2016	Sorghum Conversion Program lines and inbred checks	65	Texas A&M AgriLife Research Farm, Burleson County, Texas	*F. thapsinum and C. lunata*
Prom et al., 2017	US Breeding lines and hybrids, Senegalese lines	23	Agronomic Research Stations in Bambey, Senegal, West Africa	*F. thapsinum*
Cuevas et al., 2019a	Sorghum Association Panel	331	USDA– ARS Tropical Agriculture Research Station at Isabela and Mayaguez, PR	*Curvularia lunata, Fusarium semitectum, and F. thapsinum*
Diatta et al., 2019	Sengaleses Institute of Agricultural Research - hybrid and parental lines	10	Darou-Pakathiar, Sinthiou-Damba, Guirigara, Sinthiou-Damba, Center and South-East of Senegal, West Africa	N/A
Nida et al., 2019	Ethiopian sorghum landrace collection, breeding lines, and released varieties	1412, 1414	Bako Agricultural Research Center, Ethiopia.	*F. proliferatum, F. graminearum, F. thapsinum, F. verticillioides, F. oxysporum, Alternaria*
Nida et al., 2021	Ethiopian sorghum landrace collection	635	Bako and Jimma Agricultural Research Centers, Western Ethiopia	*Phoma, Curvularia, Fusarium, Cladosporium, Bipolaris, Alternaria, Colletotrichum and Rhizopus*
Prom et al., 2020a	Sorghum Association Panel	377	Texas A&M AgriLife Research Farm, Burleson County, Texas	*A. alternata, Fusarium thapsinum, and Curvularia lunata*
Prom et al., 2020b	Selected lines from USDA-GRIN	47	Texas AgriLife Research Farm, Burleson County, Texas	*A. alternata alone or a mixture of A. alternata, F. thapsinum and C. lunata*

*USDA – ARS, United States Department of Agriculture – Agricultural Research Service; ICRISAT, Crops Research Institute for Semi-Arid Tropics; AICSIP, All India Co-ordinated Sorghum Improvement Project; NRCS, National Research Center for Sorghum (India).*

Many of the underlying transcription factors related to SGM resistance like Y1 and TAN1 have shown widespread transcriptional regulation of a multitude of biochemical compounds. Orthologous genes such as *P1* of maize demonstrate both similar and dissimilar functions, as divergence of orthologous genes can be significant even in species such as maize and sorghum with a close evolutionary relationship (Zhang et al., 2017a). Consequently, while functional characterization and enhanced understanding of head molds in other crops such as maize and wheat demonstrate a framework for hypotheses development in sorghum, extrapolation must be approached with caution when researching the transcriptional and metabolic responses of SGM.

Many additional topics related to the understanding of the SGM disease complex suffer from a lack of general knowledge in sorghum. Included within this group is mycotoxin detoxification *in planta*, knowledge of the contribution of heterosis to host plant resistance, changes in host response through various physiological growth stages, relationships between the individual pathogens in the fungal makeup of SGM, and the effects of fungal endophytes on host resistance. Fungal endophytes have demonstrated the ability to reprogram the host transcriptome and metabolome to favor secondary metabolism in ryegrass (Dupont et al., 2015). While similar systemic effects have not been shown in sorghum, a trend such as this would result in increased host favoritism of the many components of the flavonoid pathway that are related to SGM resistance, hypothetically resulting in increased SGM resistance at a potential cost of yield due to a change in photosynthate partitioning. While few endophyte-related host systemic changes have been shown in relation to sorghum host resistances, localized biocontrol of SGM pathogens by fungal endophytes has been demonstrated (Rajini et al., 2020). Rajini et al. (2020) showed biocontrol effects were demonstrated by 26 endophytic isolates on common SGM complex pathogens: *Fusarium thapsinum*, *Epicoccum sorghinum*, *Alternaria alternata*, and *Curvularia lunata*. This study by Rajini et al. (2020) demonstrates the fungal endophyte potential for localized control, however, characterization of host systemic changes caused by the presence of fungal endophytes requires increased research to understand the potential effects on sorghum biotic resistance.

SGM is poised to increase in relevance as regions boasting warm temperatures and humidity increases due to climate change. For this reason, increased efforts are required to understand the plasticity of the grain mold complex between environments, how this affects the fungal hierarchy, and ultimately how these changes affect SGM pathogenicity on sorghum as a host. While the use of individual pathogens to isolate host response is critical to research, understanding the relationships within the disease complex is critical to understanding SGM and finding sources of resistance adapted to SGM complexes native to specific regions in international germplasm. Maintaining grain quality as a food and feed and increasing SGM resistance are both one in the same. Achieving this objective is achievable largely in part to sorghums abundant phenolic profile. However, due to the intricate and changing makeup of the SGM disease complex, continued research ventures into SGM show both notable obstacles and great potential for sorghum improvement in increased resilience, quality, and yield.

## Author Contributions

AA and AW drafted the manuscript. RB is the professor advisor who oversaw the creation of the manuscript and provided final edits. All authors contributed to the article and approved the submitted version.

## Conflict of Interest

The authors declare that the research was conducted in the absence of any commercial or financial relationships that could be construed as a potential conflict of interest.
